# Identification of Potential Virulence Factors in the Model Strain *Acinetobacter baumannii* A118

**DOI:** 10.3389/fmicb.2019.01599

**Published:** 2019-07-23

**Authors:** Maria S. Ramirez, William F. Penwell, German M. Traglia, Daniel L. Zimbler, Jennifer A. Gaddy, Nikolas Nikolaidis, Brock A. Arivett, Mark D. Adams, Robert A. Bonomo, Luis A. Actis, Marcelo E. Tolmasky

**Affiliations:** ^1^Department of Biological Science, Center for Applied Biotechnology Studies, California State University, Fullerton, Fullerton, CA, United States; ^2^Department of Microbiology, Miami University, Oxford, OH, United States; ^3^Laboratorio de Bacteriología Clínica, Departamento de Bioquímica Clínica, Facultad de Farmacia y Bioquímica, Hospital de Clínicas “José de San Martín”, Buenos Aires, Argentina; ^4^Department of Medicine, Vanderbilt University Medical Center, Nashville, TN, United States; ^5^Department of Genetics, School of Medicine, Case Western Reserve University, Cleveland, OH, United States; ^6^Departments of Pharmacology and Molecular Biology and Microbiology, Louis Stokes Cleveland Veterans Affairs Medical Center, School of Medicine, Case Western Reserve University, Cleveland, OH, United States

**Keywords:** *Acinetobacter*, virulence factors, pathogenicity, hospital infection, community infection, ESKAPE

## Abstract

*Acinetobacter baumannii* A118, a strain isolated from the blood of an infected patient, is naturally competent and unlike most clinical strains, is susceptible to a variety of different antibiotics including those usually used for selection in genetic manipulations. These characteristics make strain A118 a convenient model for genetic studies of *A. baumannii*. To identify potential virulence factors, its complete genome was analyzed and compared to other *A. baumannii* genomes. *A. baumannii* A118 includes gene clusters coding for the acinetobactin and baumannoferrin iron acquisition systems. Iron-regulated expression of the BauA outer membrane receptor for ferric-acinetobactin complexes was confirmed as well as the utilization of acinetobactin. *A. baumannii* A118 also possesses the *feoABC* genes, which code for the main bacterial ferrous uptake system. The functionality of baumannoferrin was suggested by the ability of *A. baumannii* A118 culture supernatants to cross feed an indicator BauA-deficient strain plated on iron-limiting media. *A. baumannii* A118 behaved as non-motile but included the *csuA/BABCDE* chaperone-usher pilus assembly operon and produced biofilms on polystyrene and glass surfaces. While a known capsular polysaccharide (K) locus was identified, the outer core polysaccharide (OC) locus, which belongs to group B, showed differences with available sequences. Our results show that despite being susceptible to most antibiotics, strain A118 conserves known virulence-related traits enhancing its value as model to study *A. baumannii* pathogenicity.

## Introduction

*Acinetobacter baumannii* infections used to be rare a few decades ago (Hartstein et al., 1988). However, its importance as an opportunistic human pathogen kept increasing and it is now responsible for a growing number of community and nosocomial infections including bacteremia, urinary tract infections, wound infections, meningitis, and pneumonia (Maragakis and Perl, 2008; Zurawski et al., 2012; McConnell et al., 2013; Harding et al., 2018). It mainly affects compromised patients but it has also been identified as an important causative agent of infections in wounded military personnel (Peleg et al., 2008; Petersen et al., 2011; Arivett et al., 2016). The success as an opportunistic pathogen, especially in hospital environments is due to its ability to resist desiccation and persist in the most diverse hospital locations combined with a growing resistance to disinfectants and major antibacterials (Jawad et al., 1998; Dijkshoorn et al., 2007; Perez et al., 2007; Peleg et al., 2008; Rodriguez-Bano and Bonomo, 2008; Farrow et al., 2018). Furthermore, the problematic nature of *A. baumannii* infections is enhanced by its acquisition of resistance to carbapenems, which now exceed 90% in some geographical regions with a mortality rate of about 60% (Isler et al., 2018). Recent studies identified pathogenic and resistance islands (Fournier et al., 2006; Smith et al., 2007; Post et al., 2010; Krizova et al., 2011) as well as several potential virulence factors (Harding et al., 2018) such as iron and other micronutrients uptake (Zimbler et al., 2009; Gaddy et al., 2012; Moore et al., 2014; Penwell et al., 2015), motility (Mussi et al., 2010; Eijkelkamp et al., 2013; Wood et al., 2018), production of cytotoxic and protection factors (Russo et al., 2010; Jin et al., 2011), and adhesion and biofilm formation on abiotic and biotic surfaces (Gaddy et al., 2009; Longo et al., 2014).

We have described *A. baumannii* A118, a naturally competent isolate obtained from blood of an infected patient, that is susceptible to numerous antibiotics including those commonly used in molecular genetics (Ramirez et al., 2010, 2011; Traglia et al., 2014). We proposed that these characteristics make this strain a convenient experimental model studying the pathobiology of this relevant human pathogen. Accordingly, *A. baumannii* A118 has been used in numerous studies to assess the efficiency of metal/ionophore complexes to override aminoglycoside resistance (Lin et al., 2014), to understand the effect of serum albumin in competency stability (Traglia et al., 2016; Quinn et al., 2018) and acquisition of mobile genetic elements (Ramirez et al., 2012; Domingues et al., 2018), the role of osmolarity in uptake of exogenous DNA (Domingues et al., 2019), and in the discovery of a novel bacteriophage (Turner et al., 2016). The growing number of research groups utilizing *A. baumannii* A118 as model makes characterizing this strain in greater detail desirable. In this work we focus our analysis in characteristics associated to *Acinetobacter* pathogenicity and virulence.

## Materials and Methods

### Bacterial Strains, Genomes, and Culture Conditions

*Acinetobacter baumannii* A118 was isolated from the bloodstream of an infected patient in an intensive care unit (Merkier and Centron, 2006; Ramirez et al., 2010). *Escherichia coli* TOP10 (Invitrogen, San Diego, CA, United States) was used as host in DNA recombinant cloning. *A. baumannii* ATCC 17978, ATCC 19606^T^ (Bouvet and Grimont, 1986) and the isogenic ATCC 19606^T^ s1 (BasD^–^) and t6 (BauA^–^) derivatives (Dorsey et al., 2004) were used in bioassays to test acinetobactin production and utilization, respectively. Iron-rich and iron-limiting conditions were attained by supplementing the growth media with 100 μM FeCl_3_ or 100 μM 2,2′-dipyridyl (DIP), respectively.

The presence or absence of specific coding regions in the genome sequence of *A. baumannii* A118 (GenBank Accession Number AEOW00000000) (Ramirez et al., 2011) was determined using BLAST (Altschul et al., 1990). BLASTp searches were performed with A minimum value of 30% amino acid identity, 70% coverage and a minimum *e*-value of 1 × 10^–5^. The reference genomes for sequence comparison were ATCC 17978 (Accession Number CP000521), ATCC 19606 (Accession Number ACQB00000000.1), and ACICU (Accession Number NC 010611) (Supplementary Table 1). For K Locus and OC Locus sequence analysis, we used the most related genetic structures in GenBank Database (Supplementary Table 1).

### Production and Utilization of Acinetobactin

Bioassays to test production or utilization of siderophore were carried out as previously (Dorsey et al., 2004). Briefly, supernatants from cultures using succinate medium were sterilized by filtration and spotted on filter disks placed on L agar plates containing 225 μM DIP seeded with the ATCC 19606^T^ t6 strain, which does not produce the acinetobactin receptor protein BauA. The plates were incubated for 24 h at 37°C, growth halos around the filters were an indication of production of a siderophore different from acinetobactin. The presence of the outer membrane protein BauA in *A. baumannii* strains was determined by western blotting with anti-BauA polyclonal antiserum using total lysates of bacterial cells cultured under iron-limiting or iron-rich conditions as described before (Dorsey et al., 2004). Briefly, total proteins from cells cultured under iron-rich or iron-limiting conditions were separated by SDS–PAGE using 12.5% polyacrylamide gels, transferred to a nitrocellulose membrane, and incubated in the presence of anti-BauA serum. The immunocomplexes were detected by chemiluminescence using HRP-labeled protein A (Dorsey et al., 2004).

### Cell Motility and Biofilm Assays

Cell motility was assessed using semi-solid plates containing 0.3% agarose as described before (Mussi et al., 2010). The plates were inoculated on the surface with bacteria using flat-ended sterile wooden sticks or depositing 0.003 ml of LB cultures grown to an OD_600_ of 0.3. Plates were incubated for 24 h at 24°C or 37°C in the dark or under blue light (emission peak centered at 462 nm) emitted by nine-LED (light-emitting diode) arrays with an intensity of 10 to 20 μmol photons/m^2^/s. Biofilms formed on the walls of polystyrene or glass tubes were stained with crystal violet, visually inspected and quantified after elution of the stain as previously described (Tomaras et al., 2003). The amount of biofilm formed by each sample was normalized to its total biomass, which was determined by measuring the OD_600_ of duplicate cultures as described before (Tomaras et al., 2003). Triplicate assays were done at least three times using fresh samples each time.

### Infection Assays

Randomly chosen *Galleria mellonella* larvae were injected with 1 × 10^5^ *A. baumannii* cells resuspended in sterile phosphate-buffered saline (PBS), or with PBS as negative control. After injection, the larvae were incubated at 37°C in the dark, and killing was assessed at 24-h intervals over 6 days. Caterpillars were considered dead and removed from the study if they displayed no response to probing. The results of the trial were omitted if more than two deaths occurred in the control groups. The experiments were repeated six times using 10 larvae per experimental group, and the resulting survival curves were plotted using the Kaplan-Meier method. *P-*values < 0.05 are considered statistically significant.

## Results and Discussion

*Acinetobacter baumannii* A118 is characterized for its susceptibility to a variety of antimicrobial agents such as ceftazidime, cefepime, piperacillin, minocycline, amikacin, gentamicin, sulfamethoxazole-trimethoprim, imipenem, meropenem, and ciprofloxacin (Ramirez et al., 2010). This characteristic makes it more suitable as a model for genetic analysis than most other isolates that exhibit multiresistance to antibiotics usually used for selection in a variety of techniques. To facilitate its use as model of infection we identify the presence of genes and functions previously associated with *A. baumannii* pathogenicity. The results discussed in this article are summarized in Table 1.

**TABLE 1:** Characteristics of *A. baumannii* strains.

	***A. baumannii***		
	**A118**	**ATCC 19606^T^**	**ATCC 17978**
IUS – Acinetobactin	+	+	+
IUS – Baumannoferrin	+	−	+
IUS – Fimsbactin	−	−	+
Heme cluster 1	+	+	+
Heme cluster 2	−	−	−
IUS – FeoABC	+	+	+
Biofilm	+	−	+
Motility	+	−	+
Capsular polysaccharide – locus K^1^	PSgc8	PSgc9	PSgc9
Capsular polysaccharide – locus OC^2^	New	OCL1	OCL2

*Plus signs denote functional systems. ^1^According to the classification described in Hu et al. (2013). ^2^According to the classification described in Kenyon et al. (2014).*

### Iron Uptake Systems (IUSs)

**FIGURE 1:**
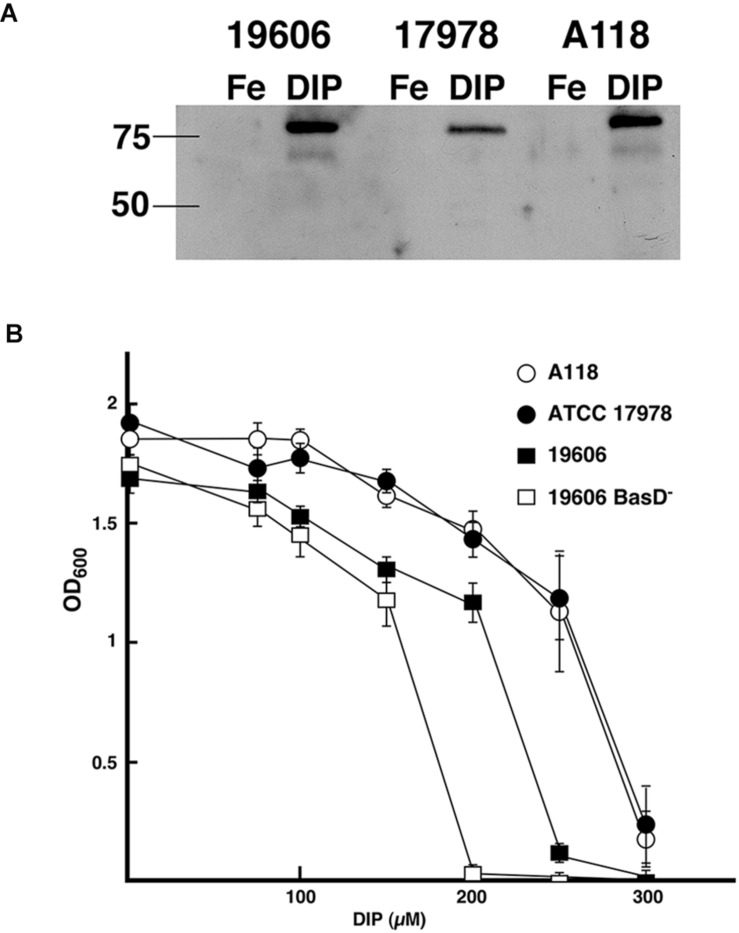
Acinetobactin iron uptake system. **(A)** Synthesis and regulation of BauA. Total proteins from *A. baumannii* ATCC 19606^T^, ATCC 17978, and A118 cultured under iron-rich (100 μM FeCl_3_) and iron-limiting (100 μM DIP) conditions were separated on a 12.5% SDS–PAGE, transferred onto nitrocellulose, and probed with anti-BauA serum. The “Precision” molecular-mass protein markers (Bio-Rad) were detected with StrepTactin-HRP conjugate. The positions of the 50- and 75-kDa protein markers are shown on the left side to the gel. 19606, ATCC 19606^T^; 17978, ATCC 17978; Fe, iron-rich conditions; DIP, iron-limiting conditions. **(B)** Bacterial growth under iron limiting conditions. The strains were cultured overnight at 37°C in L broth containing increasing concentrations of the iron chelator DIP.

A non-specific defense system of vertebrate animals against bacterial infection is the chelation of iron by high-affinity iron-binding proteins such as transferrin and lactoferrin (Crosa and Walsh, 2002). Consequently, bacteria have evolved very efficient iron-acquisition systems to scavenge iron from the iron-binding proteins of their hosts. These systems are varied and include synthesis of a siderophore (a high-affinity ferric iron chelator) that uptakes iron and mediates its internalization through a specific energy-dependent transport system (Crosa and Walsh, 2002; Di Lorenzo and Stork, 2014; Li and Ma, 2017), production of outer membrane receptors that recognize lactoferrin, transferrin, heme or non-indigenous siderophores (Koster, 2005; Antunes et al., 2011; Di Lorenzo and Stork, 2014; Huang and Wilks, 2017), or direct binding and transport of ferrous iron (Lau et al., 2016). The siderophore-mediated iron uptake systems identified in *A. baumannii* strains are those that utilize acinetobactin (Echenique et al., 1992; Actis et al., 1993, 1999), baumannoferrin (Penwell et al., 2015), or fimsbactins, which are represented by a family of six related chatechol/hydroxamate compounds (Proschak et al., 2013). Since the acinetobactin system, originally described in the ATCC 19606^T^ strain (Dorsey et al., 2004; Mihara et al., 2004), is the most widespread among *A. baumannii* isolates we identified the presence of the gene cluster as well as *entA* and *entB* orthologs, which code for functions required for the biosynthesis of the acinetobactin precursor 2,3-dihydroxybenzoic acid (Penwell et al., 2012). We also carried out assays to identify the regulated expression of BauA, the outer membrane receptor of ferric-acinetobactin complexes (Figure 1A), as well as the growth of *A. baumannii* A118 at increasing concentrations of DIP (Figure 1B). The levels and iron regulation of BauA in *A. baumannii* A118 were similar to those in the control strains ATCC 19606^T^. Both strains showed higher production of this protein as compared to the levels detected in strain ATCC 17978 (Figure 1A). However, comparison of growth of all three strains showed that while the OD_600_ levels reached by strains A118 and ATCC 17978 at increasing concentrations of DIP were nearly identical, strain ATCC 19606^T^ proved to be more sensitive to iron chelation, the growth of which was completely inhibited at a lower DIP concentration when compared with the two other tested strains (Figure 1B). The *A. baumannii* ATCC 19606^T^ s1 mutant, deficient in production of acinetobactin due to lack of the biosynthetic enzyme BasD was inhibited at the lowest DIP concentration of all four strains tested (Figure 1B). The ability of the strains A118 and ATCC 17978 to grow at higher DIP concentrations when compared to strain ATCC 19606^T^ could be due to the presence/expression of one or more additional iron acquisition systems or to a higher production of acinetobactin. It is known that *A. baumannii* ATCC 19606^T^ does not possess the genes coding for a fimsbactin siderophore and cannot express a functional baumanoferrin iron uptake system in spite of including the cognate gene cluster. This is supported by the observation that an ATCC 19606^T^ acinetobactin biosynthetic mutant cannot grow in the presence of DIP (Dorsey et al., 2004; Penwell et al., 2012, 2015). In addition to the acinetobactin gene cluster, *A. baumannii* ATCC 17978 includes the cluster coding for fimsbactin and baumannoferrin iron-uptake systems (Antunes et al., 2011; Eijkelkamp et al., 2011). Thus, the higher iron proficiency can be explained by the expression of one or both of these systems. Analysis of the *A. baumannii* A118 genome showed that it possesses the baumannoferrin gene cluster but not that one coding for a fimsbactin siderophore iron uptake system. To determine if besides the acinetobactin system, *A. baumannii* A118 expresses an additional iron uptake system we conducted a siderophore-utilization bioassays using the ATCC 19606^T^ t6 BauA-deficient strain, which cannot use acinetobactin to grow under iron-chelated conditions. Table 2 shows that the *A. baumannii* ATCC 19606^T^ t6 BauA-deficient indicator strain was able to grow around the disks spotted with supernatants obtained from cultures of strains ATCC 17978 and A118 under iron-limiting conditions. These results strongly suggest that *A. baumannii* A118 secretes an additional siderophore that, given the presence the appropriate gene cluster, it most probably is baumannoferrin. However, further chemical assays are needed to prove that A118 produces baumanoferrin.

**TABLE 2:** Siderophore utilization bioassays.

**Crossfeeding sample**	**Halo around the filter disks (mm)**
H_2_O	0
FeCl_3_	13.25 ± 0.96
ATCC 19606^T^ Sp	0
ATCC 19606^T^ s1 (BasD^–^) Sp	0
ATCC 17978 Sp	15.75 ± 1.71
A118 Sp	13.75 ± 2.22

*Strains to be tested were cultured in succinate medium for 24 h at 37°C. Culture supernatants (Sp), obtained by filter sterilization, were spotted onto filter disks that were placed on L agar plates containing 225 μM DIP and seeded with the reporter *A. baumannii* ATCC 19606^*T*^ t6 strain deficient in the production of the BauA outer membrane acinetobactin receptor. Halos were measured after incubation for 24 h at 37°C. H_2_O or FeCl_3_ were spotted as negative and positive controls, respectively.*

A search for genetic determinants of a heme-acquisition system showed that *A. baumannii* A118 posseses the heme uptake cluster 2 (Antunes et al., 2011). There is at least one report proposing that heme contributes to overcoming the iron limitation caused by the host’s high affinity iron binding proteins (de Leseleuc et al., 2014). *A. baumannii* A118 also includes the *feoABC* genes, which code for the main bacterial Feo ferrous uptake system (Cartron et al., 2006). The cluster specifies three proteins, FeoA of unknown function, the repressor FeoC, and FeoB that participates in translocation of iron across the inner membrane. A recent study showed that although the Feo system enhances growth in heat-inactivated human serum and contributes to resistance to human serum, it is not essential for virulence (Runci et al., 2019).

### Biofilm Formation

**FIGURE 2:**
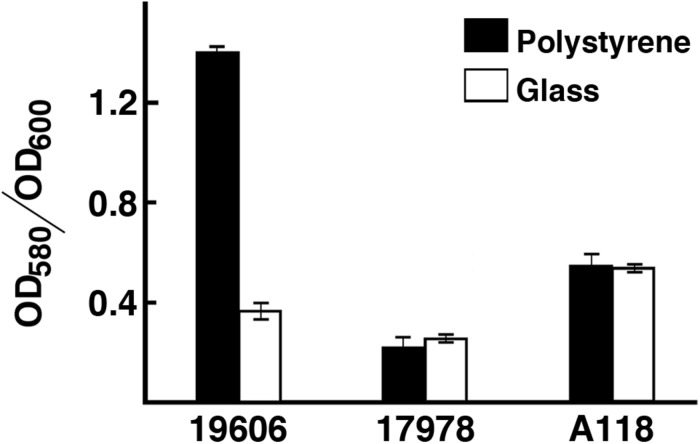
Production of biofilm. Quantification of biofilm formation on polystyrene and glass tubes by *A. baumannii* strains ATCC 19606^T^, ATCC 17978, and A118 after static incubation at 37°C. Quantification was carried out as described in “Materials and Methods.”

The ability of *A. baumannii* strains to form biofilms on abiotic surfaces (Tomaras et al., 2003) is partially or totally responsible for their persistence in different environments such as the surface of medical devices and other common artifacts like furniture, linens, soap dispensers, phones and computer keyboards (Hartstein et al., 1988, 1990; Neely, 2000; Villegas and Hartstein, 2003; Borer et al., 2005; Espinal et al., 2012). Comparative analysis of the *A. baumannii* A118 nucleotide sequence with *A. baumannii* genomes showed that the *csuA/BABCDE* chaperon-usher pilus assembly system is present in the A118 strain and the identities of the deduced amino acid sequence of the products of the cognate open reading frames is 96% or higher when compared to those from strain ATCC 17978. Standard crystal violet biofilm assays using polystyrene tubes showed that *A. baumannii* A118 formed a distinctive biofilm ring on the surface of the tubes as it was described for ATCC 19606^T^ cells (Tomaras et al., 2003). Control assays showed a stronger ring in the case of the strain ATCC 19606^T^ while significantly reduced biofilm was formed by strain ATCC 17978. Quantification of total biofilms formed on polystyrene and glass showed that strain A118 produced a significant amount of biofilm on glass and polystyrene surfaces (Figure 2). It was of interest that while the biofilm mass was higher for strain A118 than for strain ATCC 19606^T^ on glass, the opposite was true for biofilm formed on plastic (Figure 2). Although the molecular and cellular bases of these differences are unknown, variations in the amount of biofilms formed by different strains on the same or different types of surfaces seem to be a common property among *A. baumannii* clinical isolates (McQueary and Actis, 2011; Runci et al., 2017; Quinn et al., 2018; Wood et al., 2018).

### Motility

**FIGURE 3:**
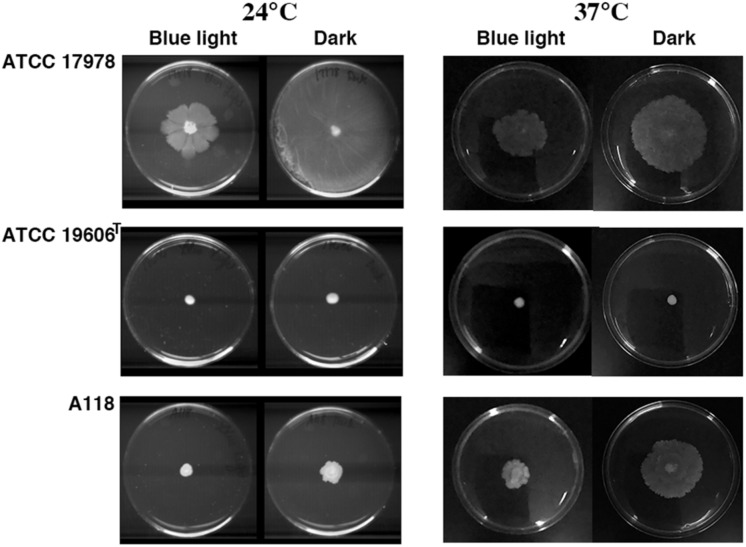
Surface motility on a semi-solid surface. Cells were inoculated on the surface of motility plates and incubated overnight at 24°C or 37°C in the dark or under blue light.

*Acinetobacter baumannii* lacks flagellum and therefore cannot show flagellum-mediated swimming motility (Towner et al., 1991). However, it has been shown that *Acinetobacter* strains are capable of motility on semi-solid agar (Barker and Maxted, 1975; Mussi et al., 2010; Eijkelkamp et al., 2011; Quinn et al., 2018; Wood et al., 2018), and the movement is dependent on the concentration of iron in the environment and possibly on other stress related factors (Eijkelkamp et al., 2011; Quinn et al., 2018; Wood et al., 2018). Furthermore, it has been shown that at 24°C strain ATCC 17978 shows low or no signs of motility when growing exposed to light but this situation is reversed when growing in the dark (Mussi et al., 2010; Figure 3). This regulation depends on the expression of BlsA, a photoreceptor and transcriptional regulator (Mussi et al., 2010), and the newly identified PrpABCD type I pilus assembly system (Wood et al., 2018). In contrast, as it was shown before, ATCC 17978 cells displayed comparable surface motility independently of illumination when cultured at 37°C (Mussi et al., 2010). Analysis of *A. baumannii* A118 showed that this strain was unable to exhibit motility under blue light at 24°C but it showed slight motility in the dark (Figure 3). This motility was more evident at 37°C in the dark although cells displayed a reduced motility when cultured at this temperature under illumination (Figure 3). *A. baumannii* ATCC 19606^T^ did not show motility in any of the assayed conditions (Figure 3). Analysis of the *A. baumannii* A118 genome showed the presence of all the genes associated with a type IV pilus system (T4P), known to play a role in *A. baumannii* twitching motility and surface-associated. Taken together, these results indicate that *A. baumannii* A118 is motile under the right conditions and that light and temperature affect the motility responses of different *A. baumannii* strains by mechanisms that remain to characterized.

### Locus K and Locus OC

**FIGURE 4:**
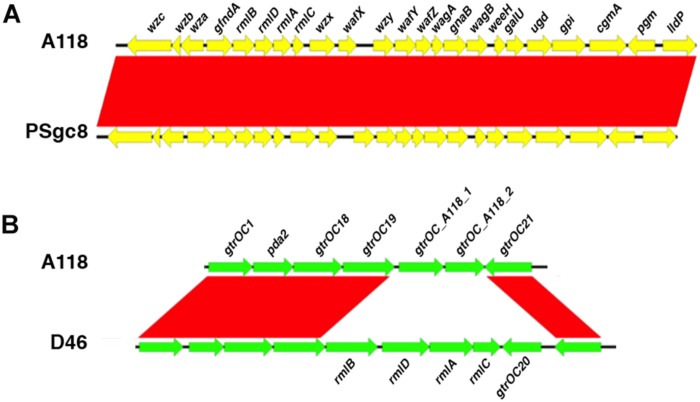
K and OC loci comparisons. **(A)** Comparison of the K loci from *A. baumannii* strains A118 and PSgc8 (Accession Number LUH5538). Comparison was performed using BLAST (version 2.2.31) and the graphic representation was generated using the software EasyFig (version 2.1). Proteins coded for by the genes are: *wzc*, Tyrosine-protein kinase; *wzb*, protein-tyrosine-phosphatase; *wza*, periplasmic protein; *gnaA*, UDP-glucose/GDP-mannose dehydrogenase; *rmlB*, dTDP-D-glucose-4,6-dehydratase; *rmlD*, dTDP-4-dehydrorhamnose reductase; *rmlA*, dTDP-glucose pyrophosphorylase; *rmlC*, dTDP-4-keto-6-deoxy-D-glucose-3,5-epimerase; *wzx*, flippase; *wafX*, glycosyltransferase; *wzy*, O antigen polymerase; *wafY*, glycosyltransferase; *wafZ*, glycosyltransferase; *wagA*, acetyltransferase; *gnaB*, NAD-dependent epimerase/dehydratase; *wagB*, glycosyl transferase; *weeH*, glycosyltransferase; *galU*, UDP-glucose pyrophosphorylase; *ugd*, UDP-glucose 6-dehydrogenase; *gpi*, glucose-6-phosphate isomerase; *cgmA*, cyclic beta-1,2-glucan modification transmembrane protein; *pgm*, phosphoglucomutase; *lldP*, L-lactate permease. **(B)** Comparison of the OC loci from *A. baumannii* strains A118 and D46 (Accession Number KF030679). All three strains, D46, ZW85-1 (Accession Number CP006768), and 6200 (Accession Number CP010397), have identical OC loci. Proteins coded for by the genes are: *gtrOC1*, glycosyltransferase; *pda2*, polysaccharide deacetylase; *gtrOC18*, glycosyltransferase; *gtrOC19*, glycosyltransferase; *gtrOC20*, glycosyltransferase; *gtrOC21*, glycosyltransferase; *gtrOC_A118_1*, capsular protein, polysaccharide synthesis; *gtrOC_A118_2*, glycosyltransferase.

*Acinetobacter baumannii* strains produce a capsular polysaccharide coded for by a gene cluster known as K locus, which exhibits sequence variations among isolates (Kenyon and Hall, 2013). Another surface polysaccharide produced by this bacterium is part of the lipooligosaccharide formed by the lipid A and the inner and outer core oligosaccharides (Kenyon et al., 2014). While the inner core oligosaccharide is well conserved, the outer core is highly variable (Erridge et al., 2007). The group of genes coding for the outer core oligosaccharide are known as the OC locus. The surface carbohydrates have been linked to the virulence of *A. baumannii* in numerous studies (Russo et al., 2010; Iwashkiw et al., 2012; Geisinger and Isberg, 2015). We found the *A. baumannii* A118 K locus structure located between the *fkpA* and *lldP* genes as it has been reported for other strains (Kenyon and Hall, 2013). Nucleotide sequence comparison of this locus showed 99% identity (100% cover) with that of the strain Sv8/PSgc8 described by Hu et al. (2013); (Figure 4A). A search to identify the OC locus showed that an *A. baumannii* A118 gene cluster exhibited 98% identity (79% cover) with those present in strains D46, ZW85-1, and 6200 (Figure 4B). None of the reported versions of the OC locus was identical to that of the *A. baumannii* A118 strain. The two genes not represented in strain D46 show 100% amino acid sequence identity with those found in strain Naval-72. On the other hand, the *rmlB*, *rmlD*, *rmlA* y *rmlC* genes present in this locus in strain D46 are located in the K locus in *A. baumannii* A118 (see Figures 4A,B). According to the OC loci classification proposed by Kenyon et al. (2014), the *A. baumannii* A118 OC locus belongs to group B.

### Experimental Infections

**FIGURE 5:**
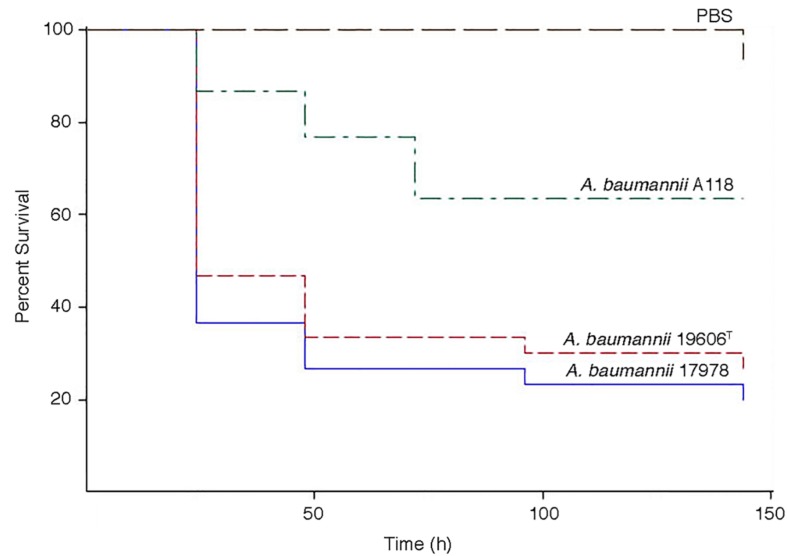
*Galleria mellonella* infection assays. Infections were carried out as described in Materials and Methods. Larvae were injected with 1 × 10^5^ cells of *A. baumannii* A118, 19606^T^, or 17978 resuspended in sterile PBS. Negative controls were injected with PBS.

The virulence of *A. baumannii* A118 was assessed using the *G. mellonella* model of infection. Figure 5 shows that larvae infections with this strain results in a significant increase in mortality as compared to the negative control. However, strain A118 showed lower virulence when compared to the *A. baumannii* ATCC 17978 and ATCC 19606T strains, both of which produced comparable larvae survival rates (Figure 5).

## Concluding Remarks

There is an urgent need to find answers to the problem of infections with bacteria belonging to the ESKAPE group (Boucher et al., 2013), which includes *A. baumannii*. Research on this bacterium presents numerous challenges due to its tremendous genetic plasticity and diversity, as well as its multiresistance to most antibiotics used for selection in the molecular biology laboratory (Ramirez et al., 2010; Wright et al., 2016; Traglia et al., 2019). We have analyzed functions related to the antibiotic resistance/susceptibility profile of the model strain *A. baumannii* A118 (Ramirez et al., 2010, 2011). Building on the characterization of this strain we show here the presence of genetic and cellular traits that could be associated to its pathogenicity. It most probably includes at least two iron uptake systems, those mediated by the siderophores acinetobactin and baumannoferrin (Dorsey et al., 2003, 2004; Mihara et al., 2004; Eijkelkamp et al., 2011). The presence of the *csu* operon is compatible with the ability of *A. baumannii* A118 to form biofilms on polystyrene and glass to levels comparable to strain ATCC 19606^T^. The K and OC loci were identified and characterized, while the K locus has been found in another isolated, the OC locus was still undescribed. Although *A. baumannii* A118 displayed more surface motility than ATCC 19606^T^ under all tested conditions, its motility seems reduced when compared to ATCC17978, particularly under illumination at 37°C. This response could be due to the absence of the gene coding for a fimbrial protein that might be involved in this response. In conclusion, *A. baumannii* A118 possesses the main potential virulence factors found in other clinical isolates reinforcing the usefulness of this strain as a model of genetic manipulation and pathogenic studies.

## Data Availability

All datasets generated for this study are included in the manuscript and/or the Supplementary Files.

## Author Contributions

MT, MR, LA, and RB conceived and designed the experiments. MR, WP, JG, GT, DZ, NN, BA, MA, LA, and MT performed the experiments. MR, WP, JG, NN, GT, DZ, BA, MA, LA, and MT analyzed the data. MA, MR, MT, and NN contributed reagents, mutant strains, materials, and analysis tools. MR and MT wrote the manuscript. MR, MT, LA, RB, and NN revised the manuscript.

## Conflict of Interest Statement

The authors declare that the research was conducted in the absence of any commercial or financial relationships that could be construed as a potential conflict of interest.

## References

[B1] Actis L. A. Smoot J. C. Barancin C. E. Findlay R. H. (1999). Comparison of differential plating media and two chromatography techniques for the detection of histamine production in bacteria. *J. Microbiol. Methods* 39 79–90. 10.1016/s0167-7012(99)00099-8 10579509

[B2] Actis L. A. Tolmasky M. E. Crosa L. M. Crosa J. H. (1993). Effect of iron-limiting conditions on growth of clinical isolates of *Acinetobacter baumannii*. *J. Clin. Microbiol.* 31 2812–2815. 8253994 10.1128/jcm.31.10.2812-2815.1993 PMC266025

[B3] Altschul S. F. Gish W. Miller W. Myers E. W. Lipman D. J. (1990). Basic local alignment search tool. *J. Mol. Biol.* 215 403–410. 10.1006/jmbi.1990.9999 2231712

[B4] Antunes L. C. Imperi F. Towner K. J. Visca P. (2011). Genome-assisted identification of putative iron-utilization genes in *Acinetobacter baumannii* and their distribution among a genotypically diverse collection of clinical isolates. *Res. Microbiol.* 162 279–284. 10.1016/j.resmic.2010.10.010 21144895

[B5] Arivett B. A. Ream D. C. Fiester S. E. Kidane D. Actis L. A. (2016). Draft genome sequences of *Acinetobacter baumannii* isolates from wounded military personnel. *Genome Announc.* 4:e773-16. 10.1128/genomeA.00773-16 27563036 PMC5000820

[B6] Barker J. Maxted H. (1975). Observations on the growth and movement of *Acinetobacter* on semi-solid media. *J. Med. Microbiol.* 8 443–446. 10.1099/00222615-8-3-443 1177290

[B7] Borer A. Gilad J. Smolyakov R. Eskira S. Peled N. Porat N. (2005). Cell phones and *Acinetobacter* transmission. *Emerg. Infect. Dis.* 11 1160–1161. 16032803 10.3201/eid1107.050221 PMC3371817

[B8] Boucher H. W. Talbot G. H. Benjamin D. K. Jr. Bradley J. Guidos R. J. Jones R. N. (2013). 10 x ′20 Progress–development of new drugs active against gram-negative bacilli: an update from the infectious diseases society of America. *Clin. Infect. Dis.* 56 1685–1694. 10.1093/cid/cit152 23599308 PMC3707426

[B9] Bouvet P. J. M. Grimont P. A. D. (1986). Taxonomy of the genus *Acinetobacter* with the recognition of *Acinetobacter baumannii* sp. *nov., Acinetobacter haemolyticus* sp. nov., *Acinetobacter johnsonii* sp. nov., and *Acinetobacter junii* sp. nov. and emended descriptions of *Acinetobacter calcoaceticus* and *Acinetobacter lwoffii*. *Int. J. Syst. Bacteriol.* 36 228–240. 10.1099/00207713-36-2-228

[B10] Cartron M. L. Maddocks S. Gillingham P. Craven C. J. Andrews S. C. (2006). Feo–transport of ferrous iron into bacteria. *Biometals* 19 143–157. 10.1007/s10534-006-0003-2 16718600

[B11] Crosa J. H. Walsh C. T. (2002). Genetics and assembly line enzymology of siderophore biosynthesis in bacteria. *Microbiol. Mol. Biol. Rev.* 66 223–249. 10.1128/mmbr.66.2.223-249.2002 12040125 PMC120789

[B12] de Leseleuc L. Harris G. Kuolee R. Xu H. H. Chen W. (2014). Serum resistance, gallium nitrate tolerance and extrapulmonary dissemination are linked to heme consumption in a bacteremic strain of *Acinetobacter baumannii*. *Int. J. Med. Microbiol.* 304 360–369. 10.1016/j.ijmm.2013.12.002 24440358

[B13] Di Lorenzo M. Stork M. (2014). Plasmid-encoded iron uptake systems. *Microbiol. Spectr.* 2:6. 10.1128/microbiolspec.PLAS-0030-2014 26104436

[B14] Dijkshoorn L. Nemec A. Seifert H. (2007). An increasing threat in hospitals: multidrug-resistant *Acinetobacter baumannii*. *Nat. Rev. Microbiol.* 5 939–951. 10.1038/nrmicro1789 18007677

[B15] Domingues S. Rosario N. Ben Cheikh H. Da Silva G. J. (2018). IS*Aba1* and Tn*6168* acquisition by natural transformation leads to third-generation cephalosporins resistance in *Acinetobacter baumannii*. *Infect. Genet. Evol.* 63 13–16. 10.1016/j.meegid.2018.05.007 29758354

[B16] Domingues S. Rosario N. Candido A. Neto D. Nielsen K. M. Da Silva G. J. (2019). Competence for natural transformation is common among clinical strains of resistant *Acinetobacter* spp. *Microorganisms* 7:30. 10.3390/microorganisms7020030 30682786 PMC6406254

[B17] Dorsey C. W. Tolmasky M. E. Crosa J. H. Actis L. A. (2003). Genetic organization of an *Acinetobacter baumannii* chromosomal region harbouring genes related to siderophore biosynthesis and transport. *Microbiol. SGM* 149 1227–1238. 10.1099/mic.0.26204-0 12724384

[B18] Dorsey C. W. Tomaras A. P. Connerly P. L. Tolmasky M. E. Crosa J. H. Actis L. A. (2004). The siderophore-mediated iron acquisition systems of *Acinetobacter baumannii* ATCC 19606 and *Vibrio anguillarum* 775 are structurally and functionally related. *Microbiology* 150 3657–3667. 10.1099/mic.0.27371-0 15528653

[B19] Echenique J. R. Arienti H. Tolmasky M. E. Read R. R. Staneloni R. J. Crosa J. H. (1992). Characterization of a high-affinity iron transport system in *Acinetobacter baumannii*. *J. Bacteriol.* 174 7670–7679. 10.1128/jb.174.23.7670-7679.1992 1447137 PMC207480

[B20] Eijkelkamp B. A. Hassan K. A. Paulsen I. T. Brown M. H. (2011). Investigation of the human pathogen *Acinetobacter baumannii* under iron limiting conditions. *BMC Genomics* 12:126. 10.1186/1471-2164-12-126 21342532 PMC3055841

[B21] Eijkelkamp B. A. Stroeher U. H. Hassan K. A. Elbourne L. D. Paulsen I. T. Brown M. H. (2013). H-NS plays a role in expression of *Acinetobacter baumannii* virulence features. *Infect. Immun.* 81 2574–2583. 10.1128/IAI.00065-13 23649094 PMC3697591

[B22] Erridge C. Moncayo-Nieto O. L. Morgan R. Young M. Poxton I. R. (2007). *Acinetobacter baumannii* lipopolysaccharides are potent stimulators of human monocyte activation via toll-like receptor 4 signalling. *J. Med. Microbiol.* 56 165–171. 10.1099/jmm.0.46823-0 17244795

[B23] Espinal P. Marti S. Vila J. (2012). Effect of biofilm formation on the survival of *Acinetobacter baumannii* on dry surfaces. *J. Hosp. Infect.* 80 56–60. 10.1016/j.jhin.2011.08.013 21975219

[B24] Farrow J. M. III Wells G. Pesci E. C. (2018). Desiccation tolerance in *Acinetobacter baumannii* is mediated by the two-component response regulator BfmR. *PLoS One* 13:e0205638. 10.1371/journal.pone.0205638 30308034 PMC6181384

[B25] Fournier P. E. Vallenet D. Barbe V. Audic S. Ogata H. Poirel L. (2006). Comparative genomics of multidrug resistance in *Acinetobacter baumannii*. *PLoS Genet.* 2:e7. 10.1371/journal.pgen.0020007 16415984 PMC1326220

[B26] Gaddy J. A. Arivett B. A. Mcconnell M. J. Lopez-Rojas R. Pachon J. Actis L. A. (2012). Role of acinetobactin-mediated iron acquisition functions in the interaction of *Acinetobacter baumannii* strain ATCC 19606T with human lung epithelial cells, *Galleria mellonella* caterpillars, and mice. *Infect. Immun.* 80 1015–1024. 10.1128/IAI.06279-11 22232188 PMC3294665

[B27] Gaddy J. A. Tomaras A. P. Actis L. A. (2009). The *Acinetobacter baumannii* 19606 OmpA protein plays a role in biofilm formation on abiotic surfaces and in the interaction of this pathogen with eukaryotic cells. *Infect. Immun.* 77 3150–3160. 10.1128/IAI.00096-09 19470746 PMC2715673

[B28] Geisinger E. Isberg R. R. (2015). Antibiotic modulation of capsular exopolysaccharide and virulence in *Acinetobacter baumannii*. *PLoS Pathog.* 11:e1004691. 10.1371/journal.ppat.1004691 25679516 PMC4334535

[B29] Harding C. M. Hennon S. W. Feldman M. F. (2018). Uncovering the mechanisms of *Acinetobacter baumannii* virulence. *Nat. Rev. Microbiol.* 16 91–102. 10.1038/nrmicro.2017.148 29249812 PMC6571207

[B30] Hartstein A. I. Morthland V. H. Rourke J. W. Jr. Freeman J. Garber S. Sykes R. (1990). Plasmid DNA fingerprinting of *Acinetobacter calcoaceticus* subspecies anitratus from intubated and mechanically ventilated patients. *Infect. Control Hosp. Epidemiol.* 11 531–538. 10.2307/30151321 2230042

[B31] Hartstein A. I. Rashad A. L. Liebler J. M. Actis L. A. Freeman J. Rourke J. W. (1988). Multiple intensive care unit outbreak of *Acinetobacter calcoaceticus* subspecies anitratus respiratory infection and colonization associated with contaminated, reusable ventilator circuits and resuscitation bags. *Am. J. Med.* 85 624–631. 10.1016/0002-9343(88)90683-3 3189366

[B32] Hu D. Liu B. Dijkshoorn L. Wang L. Reeves P. R. (2013). Diversity in the major polysaccharide antigen of *Acinetobacter baumannii* assessed by DNA sequencing, and development of a molecular serotyping scheme. *PLoS One* 8:e70329. 10.1371/journal.pone.0070329 23922982 PMC3726653

[B33] Huang W. Wilks A. (2017). Extracellular heme uptake and the challenge of bacterial cell membranes. *Annu. Rev. Biochem.* 86 799–823. 10.1146/annurev-biochem-060815-014214 28426241

[B34] Isler B. Doi Y. Bonomo R. A. Paterson D. L. (2018). New treatment options against carbapenem-resistant *Acinetobacter baumannii* infections. *Antimicrob. Agents Chemother.* 63 e01110–e01118. 10.1128/AAC.01110-18 30323035 PMC6325237

[B35] Iwashkiw J. A. Seper A. Weber B. S. Scott N. E. Vinogradov E. Stratilo C. (2012). Identification of a general O-linked protein glycosylation system in *Acinetobacter baumannii* and its role in virulence and biofilm formation. *PLoS Pathog.* 8:e1002758. 10.1371/journal.ppat.1002758 22685409 PMC3369928

[B36] Jawad A. Seifert H. Snelling A. M. Heritage J. Hawkey P. M. (1998). Survival of *Acinetobacter baumannii* on dry surfaces: comparison of outbreak and sporadic isolates. *J. Clin. Microbiol.* 36 1938–1941. 9650940 10.1128/jcm.36.7.1938-1941.1998 PMC104956

[B37] Jin J. S. Kwon S. O. Moon D. C. Gurung M. Lee J. H. Kim S. I. (2011). *Acinetobacter baumannii* secretes cytotoxic outer membrane protein a via outer membrane vesicles. *PLoS One* 6:e17027. 10.1371/journal.pone.0017027 21386968 PMC3046175

[B38] Kenyon J. J. Hall R. M. (2013). Variation in the complex carbohydrate biosynthesis loci of *Acinetobacter baumannii* genomes. *PLoS One* 8:e62160. 10.1371/journal.pone.0062160 23614028 PMC3628348

[B39] Kenyon J. J. Nigro S. J. Hall R. M. (2014). Variation in the OC locus of *Acinetobacter baumannii* genomes predicts extensive structural diversity in the lipooligosaccharide. *PLoS One* 9:e107833. 10.1371/journal.pone.0107833 25247305 PMC4172580

[B40] Koster W. (2005). Cytoplasmic membrane iron permease systems in the bacterial cell envelope. *Front. Biosci.* 10:462–477. 15574383 10.2741/1542

[B41] Krizova L. Dijkshoorn L. Nemec A. (2011). Diversity and evolution of AbaR genomic resistance islands in *Acinetobacter baumannii* strains of European clone I. *Antimicrob. Agents Chemother.* 55 3201–3206. 10.1128/AAC.00221-11 21537009 PMC3122396

[B42] Lau C. K. Krewulak K. D. Vogel H. J. (2016). Bacterial ferrous iron transport: the Feo system. *FEMS Microbiol. Rev.* 40 273–298. 10.1093/femsre/fuv049 26684538

[B43] Li Y. Ma Q. (2017). Iron acquisition strategies of *Vibrio anguillarum*. *Front. Cell Infect. Microbiol.* 7:342. 10.3389/fcimb.2017.00342 28791260 PMC5524678

[B44] Lin D. L. Tran T. Alam J. Y. Herron S. R. Ramirez M. S. Tolmasky M. E. (2014). Inhibition of aminoglycoside 6’-*N*-acetyltransferase type Ib by zinc: reversal of amikacin resistance in *Acinetobacter baumannii* and *Escherichia coli* by a zinc ionophore. *Antimicrob. Agents Chemother.* 58 4238–4241. 10.1128/AAC.00129-14 24820083 PMC4068593

[B45] Longo F. Vuotto C. Donelli G. (2014). Biofilm formation in *Acinetobacter baumannii*. *New Microbiol.* 37 119–127. 24858639

[B46] Maragakis L. L. Perl T. M. (2008). *Acinetobacter baumannii*: epidemiology, antimicrobial resistance, and treatment options. *Clin. Infect. Dis.* 46 1254–1263. 10.1086/529198 18444865

[B47] McConnell M. J. Actis L. Pachon J. (2013). *Acinetobacter baumannii*: human infections, factors contributing to pathogenesis and animal models. *FEMS Microbiol. Rev.* 37 130–155. 10.1111/j.1574-6976.2012.00344.x 22568581

[B48] McQueary C. N. Actis L. A. (2011). *Acinetobacter baumannii* biofilms: variations among strains and correlations with other cell properties. *J. Microbiol.* 49 243–250. 10.1007/s12275-011-0343-7 21538245

[B49] Merkier A. K. Centron D. (2006). bla(OXA-51)-type beta-lactamase genes are ubiquitous and vary within a strain in *Acinetobacter baumannii*. *Int. J. Antimicrob. Agents* 28 110–113. 10.1016/j.ijantimicag.2006.03.023 16844350

[B50] Mihara K. Tanabe T. Yamakawa Y. Funahashi T. Nakao H. Narimatsu S. (2004). Identification and transcriptional organization of a gene cluster involved in biosynthesis and transport of acinetobactin, a siderophore produced by *Acinetobacter baumannii* ATCC 19606T. *Microbiology* 150 2587–2597. 10.1099/mic.0.27141-0 15289555

[B51] Moore J. L. Becker K. W. Nicklay J. J. Boyd K. L. Skaar E. P. Caprioli R. M. (2014). Imaging mass spectrometry for assessing temporal proteomics: analysis of calprotectin in *Acinetobacter baumannii* pulmonary infection. *Proteomics* 14 820–828. 10.1002/pmic.201300046 23754577 PMC3883928

[B52] Mussi M. A. Gaddy J. A. Cabruja M. Arivett B. A. Viale A. M. Rasia R. (2010). The opportunistic human pathogen *Acinetobacter baumannii* senses and responds to light. *J. Bacteriol.* 192 6336–6345. 10.1128/JB.00917-10 20889755 PMC3008525

[B53] Neely A. N. (2000). A survey of gram-negative bacteria survival on hospital fabrics and plastics. *J. Burn. Care Rehabil.* 21 523–527. 10.1097/00004630-200021060-00009 11194806

[B54] Peleg A. Y. Seifert H. Paterson D. L. (2008). *Acinetobacter baumannii*: emergence of a successful pathogen. *Clin. Microbiol. Rev.* 21 538–582. 10.1128/CMR.00058-07 18625687 PMC2493088

[B55] Penwell W. F. Arivett B. A. Actis L. A. (2012). The *Acinetobacter baumannii* ent A gene located outside the acinetobactin cluster is critical for siderophore production, iron acquisition, and virulence. *PLoS One* 7:e36493. 10.1371/journal.pone.0036493 22570720 PMC3343012

[B56] Penwell W. F. Degrace N. Tentarelli S. Gauthier L. Gilbert C. M. Arivett B. A. (2015). Discovery and characterization of new hydroxamate siderophores, Baumannoferrin A and B, produced by *Acinetobacter baumannii*. *Chembiochem* 16 1896–1904. 10.1002/cbic.201500147 26235845

[B57] Perez F. Hujer A. M. Hujer K. M. Decker B. K. Rather P. N. Bonomo R. A. (2007). Global challenge of multidrug-resistant *Acinetobacter baumannii*. *Antimicrob. Agents Chemother.* 51 3471–3484. 17646423 10.1128/AAC.01464-06 PMC2043292

[B58] Petersen K. Cannegieter S. C. Van Der Reijden T. J. Van Strijen B. You D. M. Babel B. S. (2011). Diversity and clinical impact of *Acinetobacter baumannii* colonization and infection at a military medical center. *J. Clin. Microbiol.* 49 159–166. 10.1128/JCM.00766-10 21084513 PMC3020478

[B59] Post V. White P. A. Hall R. M. (2010). Evolution of AbaR-type genomic resistance islands in multiply antibiotic-resistant *Acinetobacter baumannii*. *J. Antimicrob. Chemother.* 65 1162–1170. 10.1093/jac/dkq095 20375036

[B60] Proschak A. Lubuta P. Grun P. Lohr F. Wilharm G. De Berardinis V. (2013). Structure and biosynthesis of fimsbactins A-F, siderophores from *Acinetobacter baumannii* and *Acinetobacter baylyi*. *Chembiochem* 14 633–638. 10.1002/cbic.201200764 23456955

[B61] Quinn B. Rodman N. Jara E. Fernandez J. S. Martinez J. Traglia G. M. (2018). Human serum albumin alters specific genes that can play a role in survival and persistence in *Acinetobacter baumannii*. *Sci. Rep.* 8:14741. 10.1038/s41598-018-33072-z 30282985 PMC6170387

[B62] Ramirez M. S. Adams M. D. Bonomo R. A. Centron D. Tolmasky M. E. (2011). Genomic analysis of *Acinetobacter baumannii* A118 by comparison of optical maps: identification of structures related to its susceptibility phenotype. *Antimicrob. Agents Chemother.* 55 1520–1526. 10.1128/AAC.01595-10 21282446 PMC3067174

[B63] Ramirez M. S. Don M. Merkier A. K. Bistue A. J. Zorreguieta A. Centron D. (2010). Naturally competent *Acinetobacter baumannii* clinical isolate as a convenient model for genetic studies. *J. Clin. Microbiol.* 48 1488–1490. 10.1128/JCM.01264-09 20181905 PMC2849597

[B64] Ramirez M. S. Merkier A. K. Quiroga M. P. Centron D. (2012). *Acinetobacter baumannii* is able to gain and maintain a plasmid harbouring In35 found in *Enterobacteriaceae* isolates from Argentina. *Curr. Microbiol.* 64 211–213. 10.1007/s00284-011-0052-9 22119898

[B65] Rodriguez-Bano J. Bonomo R. A. (2008). Multidrug-resistant *Acinetobacter baumannii*: eyes wide shut? *Enferm. Infecc. Microbiol. Clin.* 26 185–186. 10.1016/s0213-005x(08)72688-0 18381036

[B66] Runci F. Bonchi C. Frangipani E. Visaggio D. Visca P. (2017). *Acinetobacter baumannii* biofilm formation in human serum and disruption by gallium. *Antimicrob. Agents Chemother.* 61:e1563-16. 10.1128/AAC.01563-16 27799219 PMC5192145

[B67] Runci F. Gentile V. Frangipani E. Rampioni G. Leoni L. Lucidi M. (2019). Contribution of active iron-uptake to *Acinetobacter baumannii* pathogenicity. *Infect. Immun.* 87:e755-18. 10.1128/IAI.00755-18 30718286 PMC6434119

[B68] Russo T. A. Luke N. R. Beanan J. M. Olson R. Sauberan S. L. Macdonald U. (2010). The K1 capsular polysaccharide of *Acinetobacter baumannii* strain 307-0294 is a major virulence factor. *Infect. Immun.* 78 3993–4000. 10.1128/IAI.00366-10 20643860 PMC2937447

[B69] Smith M. G. Gianoulis T. A. Pukatzki S. Mekalanos J. J. Ornston L. N. Gerstein M. (2007). New insights into *Acinetobacter baumannii* pathogenesis revealed by high-density pyrosequencing and transposon mutagenesis. *Genes Dev.* 21 601–614. 10.1101/gad.1510307 17344419 PMC1820901

[B70] Tomaras A. P. Dorsey C. W. Edelmann R. E. Actis L. A. (2003). Attachment to and biofilm formation on abiotic surfaces by *Acinetobacter baumannii*: involvement of a novel chaperone-usher pili assembly system. *Microbiology* 149 3473–3484. 10.1099/mic.0.26541-0 14663080

[B71] Towner K. J. Bergogne-Berezin E. Fewson C. A. (1991). “Acinetobacter: portrait of a genus,” in *The Biology of Acinetobacter*, ed. Fewson C. A. (New York, NY: Plenum Press), 1–24. 10.1007/978-1-4899-3553-3_1

[B72] Traglia G. M. Chua K. Centron D. Tolmasky M. E. Ramirez M. S. (2014). Whole-genome sequence analysis of the naturally competent *Acinetobacter baumannii* clinical isolate A118. *Genome Biol. Evol.* 6 2235–2239. 10.1093/gbe/evu176 25164683 PMC4202317

[B73] Traglia G. M. Place K. Dotto C. Fernandez J. S. Montana S. Bahiense C. D. S. (2019). Interspecies DNA acquisition by a naturally competent *Acinetobacter baumannii* strain. *Int. J. Antimicrob. Agents* 53 483–490. 10.1016/j.ijantimicag.2018.12.013 30611868 PMC6456398

[B74] Traglia G. M. Quinn B. Schramm S. T. Soler-Bistue A. Ramirez M. S. (2016). Serum albumin and Ca2+ are natural competence inducers in the human pathogen *Acinetobacter baumannii*. *Antimicrob. Agents Chemother.* 60 4920–4929. 10.1128/AAC.00529-16 27270286 PMC4958237

[B75] Turner D. Wand M. E. Sutton J. M. Centron D. Kropinski A. M. Reynolds D. M. (2016). Genome sequence of vB_AbaS_TRS1, a viable prophage isolated from *Acinetobacter baumannii* strain A118. *Genome Announc.* 4:e1051-16. 10.1128/genomeA.01051-16 27738026 PMC5064099

[B76] Villegas M. V. Hartstein A. I. (2003). Acinetobacter outbreaks, 1977–2000. *Infect. Control. Hosp. Epidemiol.* 24 284–295. 10.1086/502205 12725359

[B77] Wood C. R. Ohneck E. J. Edelmann R. E. Actis L. A. (2018). A light-regulated type I pilus contributes to *Acinetobacter baumannii* biofilm, motility, and virulence functions. *Infect. Immun.* 86:e442-18. 10.1128/IAI.00442-18 29891547 PMC6105899

[B78] Wright M. S. Iovleva A. Jacobs M. R. Bonomo R. A. Adams M. D. (2016). Genome dynamics of multidrug-resistant *Acinetobacter baumannii* during infection and treatment. *Genome Med.* 8:26. 10.1186/s13073-016-0279-y 26939581 PMC4776386

[B79] Zimbler D. L. Penwell W. F. Gaddy J. A. Menke S. M. Tomaras A. P. Connerly P. L. (2009). Iron acquisition functions expressed by the human pathogen *Acinetobacter baumannii*. *Biometals* 22 23–32. 10.1007/s10534-008-9202-3 19130255

[B80] Zurawski D. V. Thompson M. G. Mcqueary C. N. Matalka M. N. Sahl J. W. Craft D. W. (2012). Genome sequences of four divergent multidrug-resistant *Acinetobacter baumannii* strains isolated from patients with sepsis or osteomyelitis. *J. Bacteriol.* 194 1619–1620. 10.1128/JB.06749-11 22374953 PMC3294875

